# The role of bacteriophages in shaping bacterial composition and diversity in the human gut

**DOI:** 10.3389/fmicb.2023.1232413

**Published:** 2023-09-19

**Authors:** Samia S. Alkhalil

**Affiliations:** Department of Clinical Laboratory Sciences, College of Applied Medical Sciences, Shaqra University, Alquwayiyah, Riyadh, Saudi Arabia

**Keywords:** phages, gastrointestinal tract, gut microbiota, interactions, diversity, lytic, lysogenic

## Abstract

The microbiota of the gut has continued to co-evolve alongside their human hosts conferring considerable health benefits including the production of nutrients, drug metabolism, modulation of the immune system, and playing an antagonistic role against pathogen invasion of the gastrointestinal tract (GIT). The gut is said to provide a habitat for diverse groups of microorganisms where they all co-habit and interact with one another and with the immune system of humans. Phages are bacterial parasites that require the host metabolic system to replicate via the lytic or lysogenic cycle. The phage and bacterial populations are regarded as the most dominant in the gut ecosystem. As such, among the various microbial interactions, the phage-bacteria interactions, although complex, have been demonstrated to co-evolve over time using different mechanisms such as predation, lysogenic conversion, and phage induction, alongside counterdefense by the bacterial population. With the help of models and dynamics of phage-bacteria interactions, the complexity behind their survival in the gut ecosystem was demystified, and their roles in maintaining gut homeostasis and promoting the overall health of humans were elucidated. Although the treatment of various gastrointestinal infections has been demonstrated to be successful against multidrug-resistant causative agents, concerns about this technique are still very much alive among researchers owing to the potential for phages to evolve. Since a dearth of knowledge exists regarding the use of phages for therapeutic purposes, more studies involving experimental models and clinical trials are needed to widen the understanding of bacteria-phage interactions and their association with immunological responses in the gut of humans.

## 1. Introduction

The gastrointestinal tract (GIT) of humans is a complex environment extending from the oral cavity to the anus. Billions of microorganisms are contained in the GIT (Sutton and Hill, 2019; Cao et al., 2022), comprising varying communities of microorganisms, which are collectively known as gut or enteric microbiota (Zuo and Ng, 2018; Sanders et al., 2021). The microbial communities in the GIT, alongside their respective genes/genomes, vary across the various components (i.e., rectum, large intestine, small intestine) and in the feces (Cao et al., 2022). The most characterized and studied gut microbiota are the bacterial species, where many of their genes have been sequenced (Cao et al., 2022). The gut microbiota has evolved with human hosts over a long period of time and is thought to provide the host with significant health benefits (Sanders et al., 2021). The production of nutrients (such as short-chain fatty acids and vitamins), the metabolism of drugs, the modulation of the immune system, and the display of an antagonistic role against pathogen invasion of the GIT are just a few examples (Shkoporov et al., 2019; Jansen and Matthijnssens, 2023). Sutton and Hill (2019) and Kirsch et al. (2021) reported that the gut serves as a habitat for a variety of microorganisms. However, variations in pH, bile salt, availability of nutrients and water, and oxygen content all work together to influence the quantity and relative diversity of the gut microbiome. Furthermore, bacteriophages (commonly referred to as phage-viruses or phages) significantly influence bacterial abundance and distribution in the gut of humans (Sutton and Hill, 2019).

The gut of infants immediately after birth is said to be free of microorganisms (Dahlman et al., 2021). However, rapid colonization of the gut by varying species of microorganisms was reported a few hours after delivery; thus, the gut becomes a habitat of great microbial diversity (Dahlman et al., 2021; Federici et al., 2021; Łobocka et al., 2021; Gummalla et al., 2023). These variations are influenced by anatomical, nutritional, pharmacological (e.g., the utilization of probiotics, laxatives, prokinetics, and antibiotics), pathological (e.g., systemic and gastrointestinal infections), and environmental (e.g., conditions in the workplace, lifestyle, family composition, and climate) factors (Scarpellini et al., 2015). Phages are among the early microbial diversity that colonizes the gut of infants after birth, as they have been isolated in stool samples of infants a few days postpartum (Dahlman et al., 2021). The composition of the phage gut is said to be very dynamic during the initial growth stages in the lives of humans with viral species turnover. This has been suggested to be the product of phages’ colonization of the human gut in a stepwise manner, owing to the different lifestyles of each individual. Temperate phages are predominantly high in situations where the biomass of bacterial species is low with a high scarcity of potential phage hosts (Dahlman et al., 2021). As time passes, bacterial species expansion throughout various components of the gut enhances virulent phage colonization. In line with this, there is an increase in the abundance of families of virulent phages such as the crAss-like and Microviridae observed later during infancy (Dahlman et al., 2021). In contrast to the gut of infants, the virome of adults is considerably more stable. Adult virome stability has been attributed to a highly persistent and abundant set of phages, which have been found to consist mainly of Microviridae and crAss-like families of phages. The aforementioned families of phages have generally been considered highly virulent and specific across human hosts with only a few if at all any shared among individuals (Dahlman et al., 2021).

The relevance and roles of phages in the GIT in terms of human health cannot be overstated. The gut microbiota’s composition and abundance can be regulated by phages. In addition, phages modulate the anti-inflammatory actions of the immune system not by just eliminating the bacterial pathogens but by interacting directly with pro-inflammatory cytokines in cells as well as reducing reactive oxygen species (ROS) overproduction (Łusiak-Szelachowska et al., 2017). By doing so, oxidative stress is being downregulated. Furthermore, the interactions of phages with lymphoid tissues associated with the gut produce an immunomodulating protective effect, which is similar to the health benefits conferred on the human body by probiotic consumption (Łusiak-Szelachowska et al., 2017). Owing to the plethora of health benefits and roles played by the microbiota of the human gut, it has become imperative to study the interactions and relationships of the gut microbiota. Especially, bacteria-phage co-existence in the human gut and the direct and indirect effect of their interactions on the overall health of humans can be understood by first understanding the lifestyle and abundance of bacteriophages in the GIT of humans.

### 1.1. Phages

**Figure 1:**
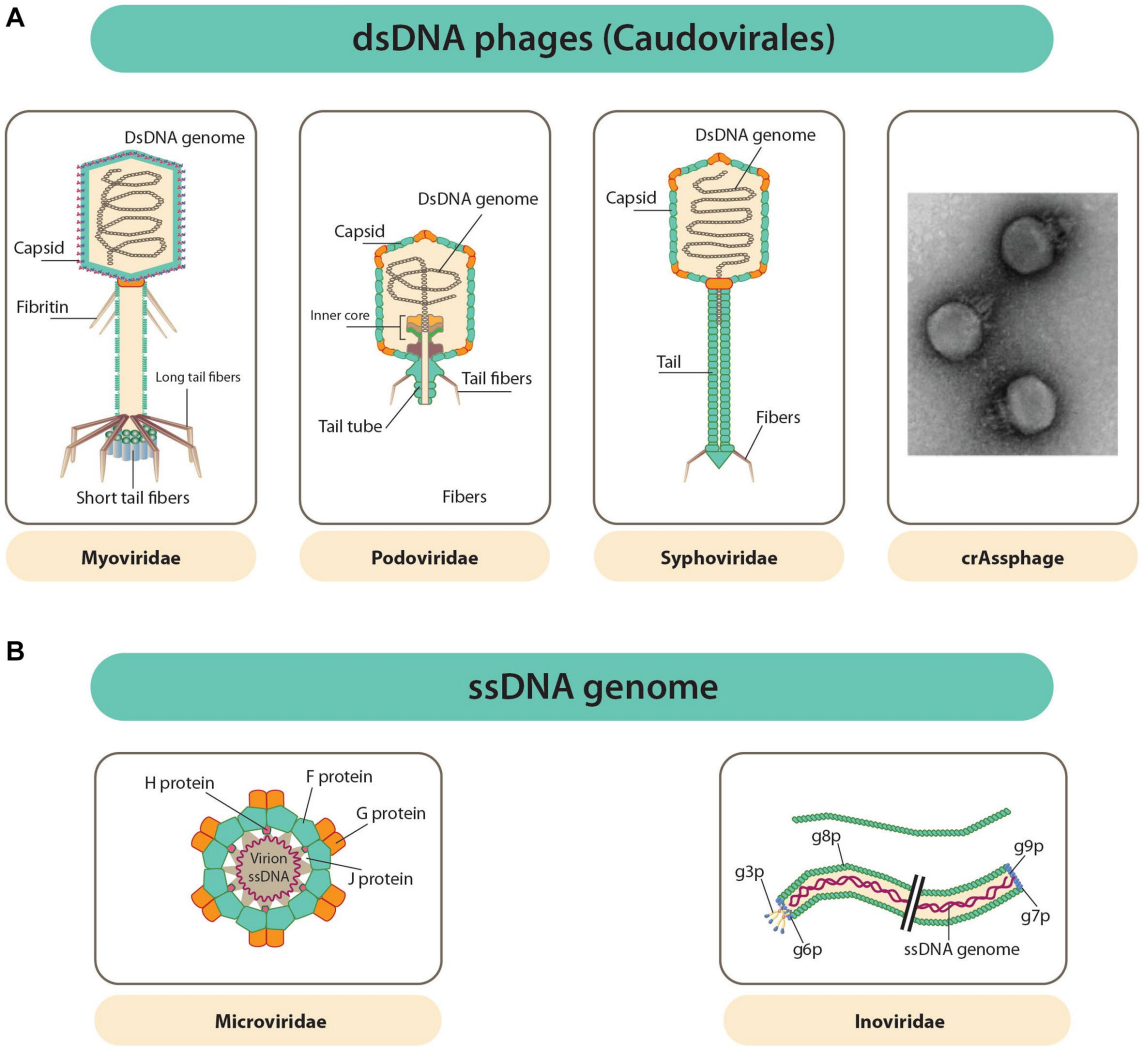
Diverse phage capsids and genomes within phages known to inhabit the GIT. **(A)** Caudovirales order dsDNA phages have capsids that is polyhedral and attached to a tail (a common feature of this order). Far right is uranyl acetate contrasted TEM image of a crAssphage (FerAss001). **(B)** ssDNA phages with capsids that are icosahedral or filamentous (Microviridae and Inoviridae in that order) (Zuppi et al., 2022).

Phages are parasitic in nature and, as such, require a host (bacteria) for their reproduction (Hannigan et al., 2018; Zuppi et al., 2022). Phages have been identified as the most diverse and abundant entity. Structurally, most phages are made up of a genome of nucleic acid packed inside the capsid (a protein shell). The capsids of phages are highly variable in terms of morphology (i.e., pleomorphic, filamentous, or polyhedral) and size (Figures 1A,B; Zuppi et al., 2022). Aside from the protein capsid, the outer layer of some phages is composed of a lipid membrane (Zuppi et al., 2022). Phages are classified based on the type of nucleic acid they possess, the morphology of their capsid, the absence or presence of a tail, and the presence or absence of an envelope (Sausset et al., 2020; Cao et al., 2022; Zuppi et al., 2022). The genetic material of phages is either made up of single-stranded (ss) or double-stranded (ds) RNA or DNA, with their genome sizes ranging from ∼3.5 kb (such as the *Escherichia coli* phage MS2 ssRNA genome) to ∼540 kb (like the LAK phages of *Prevotella*, whose genome is made up of dsDNA) (Sausset et al., 2020; Cao et al., 2022).

Although there is a great deal of variation among phages, 95% are Caudovirales (tailed, non-enveloped dsDNA phages). The Caudovirales are further traditionally differentiated into the families Podoviridae, Myoviridae, and Siphoviridae, as depicted in Figure 1A (Zuppi et al., 2022). This differentiation was based on their type of tails, but since this classification is not fully coherent with viral phylogeny, it was abandoned. Furthermore, new types of phages are constantly discovered, making classification quite difficult due to ongoing reorganization (Sausset et al., 2020). Most other phages appear as DNA viruses whose DNA is not enclosed, either as ssDNA Microviridae or dsDNA Caudovirales (Zuppi et al., 2022). The Inoviridae or filamentous ssDNA phages, which reproduce through infections that are chronic without killing the bacterial host (Figure 1B), are said to belong to a large fraction of the gut virome of humans. RNA phages are said to be absent in the gut of humans (Sausset et al., 2020). The phageome of a healthy test subject has been estimated to have active phage species between 35 and 2,800, and 50% or more of these numbers are said to be unique in each individual.

### 1.2. Structure and life cycles of bacteriophages

Phages can be found in the intestine and have been broadly distinguished into temperate and lytic phages (also known as non-temperate phages) (Sausset et al., 2020). Based on their lifestyle independent of taxonomy, there are four categories of phages, namely, (i) lytic and non-temperate, (ii) lytic and temperate, (iii) chronic and temperate, and (iv) chronic and non-temperate (Kirsch et al., 2021).

**Figure 2:**
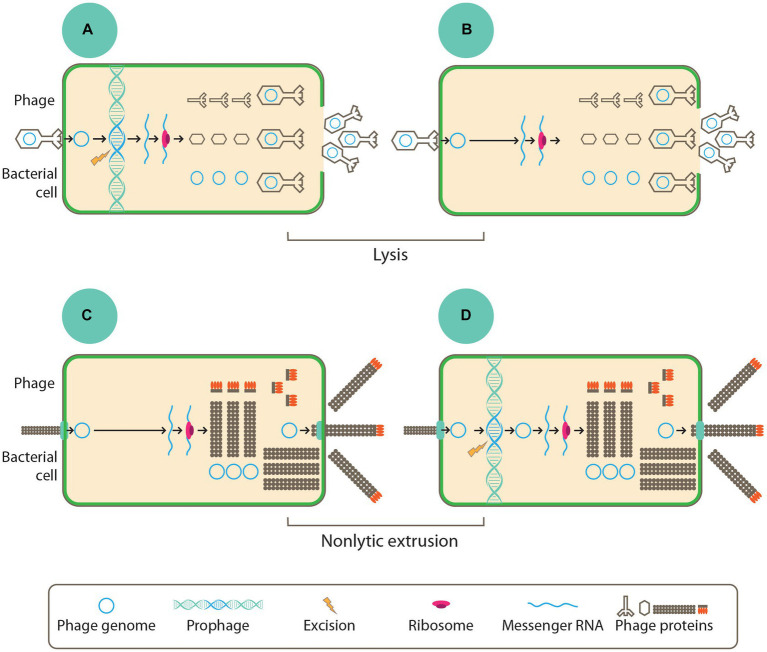
To begin any of the life style, all phages must adsorb into the host cell through the bacterial cell surface where they introduce their genome. In panels **(B,C)** replication and assemblage of new virions was done by non-temperate phages while the temperate phages as seen in panels **(A,D)** integrates their chromosome into the host chromosome where they are maintained as prophage or episome. Phage genes are early expressed where they typically contain proteins, which are involved the replication of the phage genome. The late gene expression ensures the production of phage structural proteins. Finally, virions of phages are assembled before they get released into the environment via cell lysis as depicted in panels **(A,B)** or through non-lytic extrusion in panels **(C,D)** (Kirsch et al., 2021).

Temperate phages carry out the lysogenic life cycle by integrating into the chromosomes of the bacterial hosts as extrachromosomal episomes or getting maintained as prophages. The prophages stably maintained in the chromosomes of the host can then be transmitted to the host’s progenies through vertical transmission. In adverse conditions or in response to stressors or stimuli within the host cells, the prophages are then induced to enter a lytic cycle of replication (Sausset et al., 2020), in which infectious virions are then released into the environment following the lysis of host cell as depicted in Figure 2A (Kirsch et al., 2021). However, some other phages do not follow this mode of replication since they cannot form stable lysogens. Rather, they follow the lytic replication cycle, where they cause the lysis of host cells following replication. The lytic non-temperate phages depicted in Figure 2B are generally parasitic in nature, and they also carry out horizontal gene transfer among the host population. Phages like the filamentous inoviruses, however, do not need the lysis of host cells to occur before releasing their infectious virions; these phages can cause infections that are chronic through the continuous extrusion of virions from the cell into the immediate environment, as presented in Figures 2C,D.

### 1.3. The gut phageome

The phageome of humans consists of the total phage populations, including their genomes, found throughout the GIT. The intestine of humans has been estimated to provide habitat to about 35–2,800 active phages in a gram (1 g) of feces (Spencer et al., 2022). Despite the existing gap in the taxonomy of phages, where the uncharacterized are referred to as “dark matter,” phage sequences are claimed to be the most prominent in the gut virome (Sinha and Maurice, 2019; Baaziz et al., 2022). Nonetheless, a large number of phages isolated from human intestinal virome have been reported to be DNA-encoded (Cao et al., 2022). Nevertheless, the composition of these phages is highly specific across individuals, where they comprise both lytic and temperate phages, with the former being predominant based on the viral particles sequenced as well as the gut bacteria prophages (Baaziz et al., 2022). The diversity of the gut phages, however, is dependent on some factors, which to a great extent affect the gut phageome composition.

### 1.4. Factors affecting diversity and composition of gut phages

From birth to adulthood, there are varying factors that shape and impact the diversity and composition of gut phages (Tiamani et al., 2022). These include age, diet, and the presence of diseases, among others (Sinha and Maurice, 2019; Kirsch et al., 2021).

#### 1.4.1. Age

**Figure 3:**
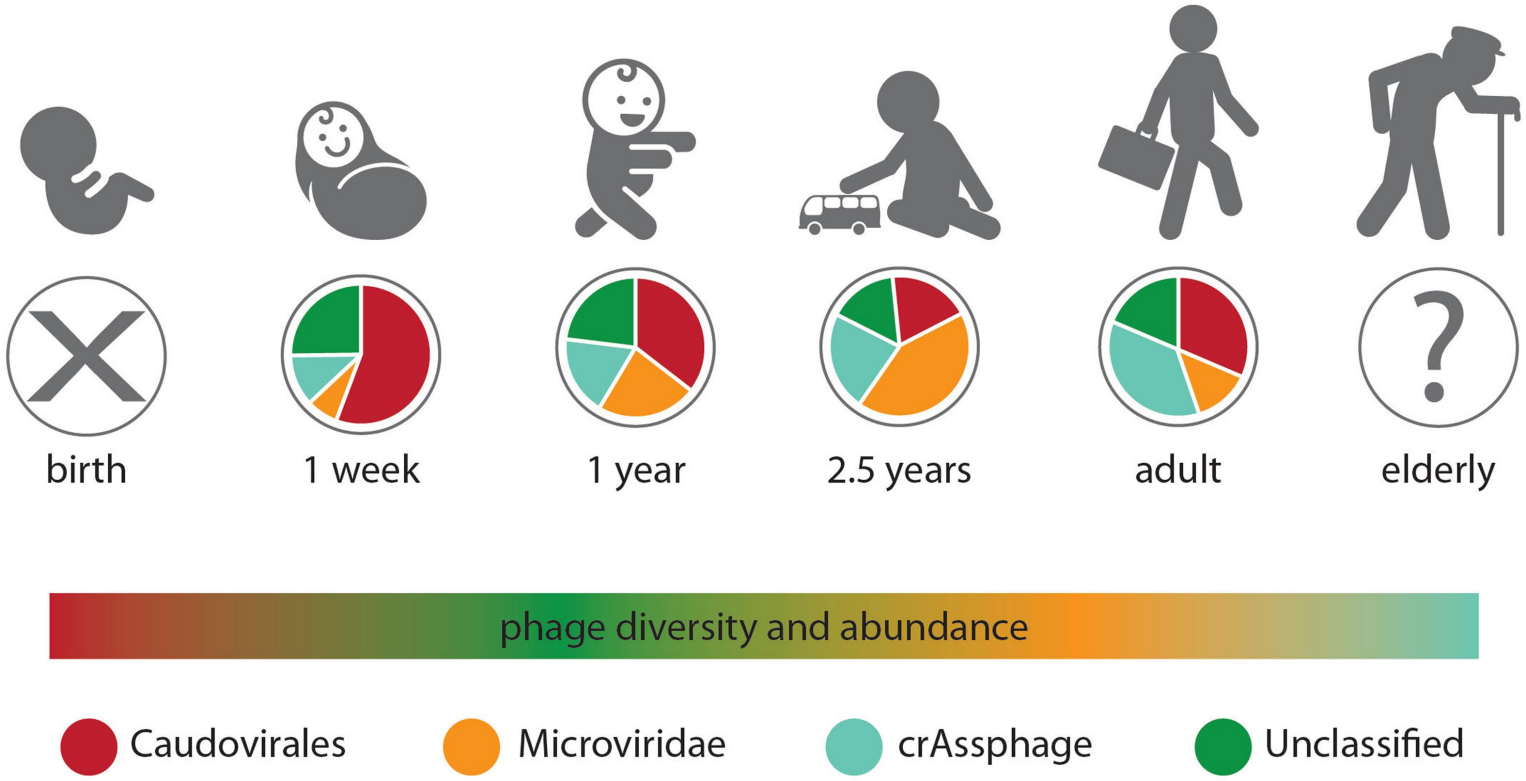
Changes in the gut phageome over the human lifetime. Pie charts represent the observed ratios of different phage groups at discrete sampling times, from birth where no endemic phages were observed to adults, while the humped line describes changes in phage diversity and abundance over time, which both peak in the weeks after birth (Townsend et al., 2021).

The gut phage composition and development have been attributed to the age of the respective individuals (Cao et al., 2022). Immediately after birth, the body of infants undergoes constant colonization by varying phage populations from various sources, such as the mother’s normal flora of the vagina (Barr, 2017; Sinha and Maurice, 2019; Kirsch et al., 2021). During infancy, an increase was reported in the abundance of phages; however, some studies have stated the opposite, where the dynamic nature of phages was reported to decrease during adolescence’s period and reach its maximum during adulthood. This eventually decreases with age (Sanders et al., 2021; Jansen and Matthijnssens, 2023). The gut phage composition of infants is said to be low but increases in abundance among Siphoviridae and Microviridae and decreases as time goes on among the Myoviridae family. Viral-like particles (VLPs) quantification of meconium (the first stool of infants) showed the presence of little or no VLPs (Figure 3). However, following one month postpartum, the VLPs numbers increased to 10^9^/g of fecal matter. Mode of delivery (i.e., cesarean or vaginal) has been identified as a confounding factor that significantly impacts the beta and alpha diversity of the infant’s gut virome within 1 year, with no significant difference observed in the bacterial diversity (Tiamani et al., 2022). There is evidence that initial phages colonizing the gut of infants are induced phages emanating from groups of bacteria that are among the first to colonize the infant’s gut, such as Firmicutes, Bacteroidetes, Actinobacteria, and Proteobacteria, which are also acquired at birth (Tiamani et al., 2022). The *Bifidobacteria* transmitted during infants’ breastfeeding, for example, has been reported to contain prophages known as bifidophages. These bifidophages belong to the Caudovirales and have been identified to dominate the phageome with little crAssphage percentage during the early life of the infant (Tiamani et al., 2022). After the colonization of the infant’s gut by *Bacteroides*, between 1 and 3 months, the crAssphages begin to appear. Similarly, the *Bifidobacteria* and bifidophages that colonize the gut of infants together with the crAssphages have shown genomic similarity up to 99% to the crAssphages identified from maternal gut sequencing (Tiamani et al., 2022).

It is worth mentioning that with age, the diversity and richness of phages decrease inversely with the diversity and richness of the bacterial community (Tiamani et al., 2022). Still, in the gut of infants, the diversity of bacteriomes and phageomes is said to be inversely relational, shifting from low bacteriome and high phageome communities within the first 4 days to a high diversity of bacteriomes and low phageome communities at the age of 2 years (Cao et al., 2022). In early childhood, however, the gut phageome-dominated Caudovirales gradually lose dominance to the Microviridae family (Townsend et al., 2021). However, during adulthood, the human gut virome is dominated by the abundant presence of crAssphages (Tiamani et al., 2022). For example, in the individual meta-analysis of viromes of the human gut, one population of crAssphages was reported to be 12% of the total samples analyzed across varying age categories globally (Kirsch et al., 2021). From the results obtained, it was deduced that the abundance of crAssphages peaks during adult life but decreases as individuals grow old. Recent evidence also supports that crAssphages infect more of the Bacteroidetes bacteria, including *Bacteroides intestinalis*, *Bacteroides thetaiotaomicron*, and *Porpyromonas* sp. (Kirsch et al., 2021).

#### 1.4.2. Diet type

Among the factors that determine or shape the gut virome of humans is the type of diet consumed by individuals (Martinez-Guryn et al., 2019; Pargin et al., 2023), although variations within individuals are the most significant differentiator of the gut virome of humans. However, convergent viromes are observed among people who consume similar diets (Pargin et al., 2023). A change in diet can produce a shift in the gut virome diversity of an individual. This occurrence has been observed in individuals who have altered their intake of alcohol, gluten, fat, and meat to become vegetarian as well as in those who have changed the frequency and time of meal consumption (Martinez-Guryn et al., 2019; Pargin et al., 2023). Although a change in diet has been reported to cause a measurable shift in the gut virome as well as impact the health of the respective individuals via the gut-brain axis. However, the exact mechanism(s) of how this virome community changes and how it is influenced by diet remains to be determined (Pargin et al., 2023). In a 4-month study that compares the influence of feeding infants breast milk compared to those fed with infant formula, it was observed that both studies differ significantly with respect to the abundance of virome.

Infants subjected to dietary interventions in the study demonstrated that a change in diet causes a change in the gut virome’s diversity and richness. Furthermore, studies have revealed that dietary ingredients commonly used, such as oregano, cinnamon, and coffee, among others, as well as medications taken orally, have the capacity for prophage inducement, which in turn can alter the composition and dynamics of the gut virome (Tiamani et al., 2022). The impact diet has on the composition of the gut phageome. It was reported that in individuals on similar diets, a large amount of variance existed between them in terms of their gut virome. These findings were challenged, and it was discovered that feeding identical diets to different individuals has the potential to shape the microbiome composition to a more similar phageome composition but not identical as earlier suggested (Mukhopadhya et al., 2019). This conflicting finding has been attributed to variation in research population size, variability, and constraints in the different subject materials and methodologies, bearing in mind current gaps limiting the study of the gut phageome (Mukhopadhya et al., 2019).

Jansen and Matthijnssens (2023) reported that individuals on similar diets tend to have a more similar composition of their gut virome, which changes to some degree whenever there is any form of dietary intervention. However, a relatively low presence of Caudoviricetes and a relatively high presence of Malgrandaviricetes phages were observed among individuals who were placed on a diet filled with high fat.

#### 1.4.3. Presence of disease(s)

The health status of individuals has also been considered one of the factors that impact the diversity of gut virome. A considerable variance in the gut virome was reported among healthy and unhealthy individuals with significant implications for the immune system (Manohar et al., 2019). Numerous reports have been made regarding the imbalance among various orders caused by varying disorders, including inflammatory bowel disease (IBD), diarrhea, metabolic disorders, diabetes, and obesity (Łusiak-Szelachowska et al., 2020). Reports have also been made regarding how difficult it is to determine whether the presence of a disease condition causes or influences a shift in gut virome diversity (Łusiak-Szelachowska et al., 2020).

Koch’s postulates, laid down by Robert Koch, are used to identify causal relationships between microorganisms and disease conditions. The metagenomic version of Koch’s postulates contributes considerably to the variation in metagenomic traits discovered between healthy individuals and those who are sick, as well as evidence that sample inoculation from a sick individual to a healthy individual induces the manifestation of the disease/infection from which the sample was collected (Łusiak-Szelachowska et al., 2020). However, in human phage research, not all of Koch’s postulates are supported. For example, phage or viral contigs significantly differ between healthy and sick individuals with only a few studies that have been performed mostly with fecal microbiota transplantation (FMT) rather than with phage inoculation (Łusiak-Szelachowska et al., 2020). More so, temperate phages are the most abundant in the human gut, thus suggesting that the microbiome of the gut is fairly stable in the GIT, suggesting a possible global establishment of the intestinal phageome. This can demonstrate a correlation between the health status of a person and the roles played by phages in maintaining the functions and structure of the GIT microbiome of humans. The maintenance of other intestinal microbiota has been suggested to be possible as a function of the stability of the phage population. Patients with Crohn’s disease (CD) and ulcerative colitis (UC) have been investigated for the roles of phages in the respective intestinal disorders. Interestingly, it was discovered that healthy phageome prevalence was altered significantly. Measuring the richness of phages in each sample revealed a large number of phage species among CD and UC patients, with a corresponding decrease in the richness of bacterial species. The inducible DNA repair system regulated by two major regulators, repressor LexA and inducer RecA (SOS response), was observed to be associated with the inflamed gut as a regulatory response against loss of diversity of phageomes, pathogenic bacteria gut, as well as prophages induction. Various environmental factors or conditions have been attributed to triggering the SOS response by the bacterial cells, such as antibiotics, ultraviolet light, or even the host immune response (Gutiérrez and Domingo-Calap, 2020).

The use of antibiotics has a potential effect that is long-lasting against the gut microbiota, and their usage in the early life of an individual has been related to a higher risk of developing various health disorders, including inflammatory bowel disease (IBD) and even asthma. Antibiotics have been demonstrated to greatly affect gut microbial diversity, such as anaerobic bacterial species that are beneficial in maintaining the gut microbiota. Antibiotics impair bacteria by disrupting essential processes or structures, thereby causing bacteriostatic or bactericidal effects (Kohanski et al., 2010). Such important species of bacteria that get impaired owing to antibiotic usage include *Clostridia*, *Bacteroides*, *Lactobacilli*, and *Bifidobacteria*, among many others. Following only 7 days of treatment with antibiotics, the diversity of microorganisms was observed to have decreased by over 25%, with the microbiota reducing to 12 from an initial 29 taxa with a 2.5-fold increase among the bacterial species, which are resistant to indiscriminate use of antibiotics (Sanders et al., 2021). However, the extent of damage caused by antibiotics to any microbial population is dependent on the types of antibiotics (Sanders et al., 2021). Duerkop (2018) reported a significant effect on the bacterial and phage communities due to the administration of antibiotics. It was suggested that the adverse effects of antibiotics caused the expression of genes responsible for the inducement and transcription of lytic genes present in the prophages, which may contribute to the lysis and eventual death of infected bacterial cells (Duerkop, 2018).

## 2. Bacteriophage-bacteria interactions of the GI tract microbiota

**Figure 4:**
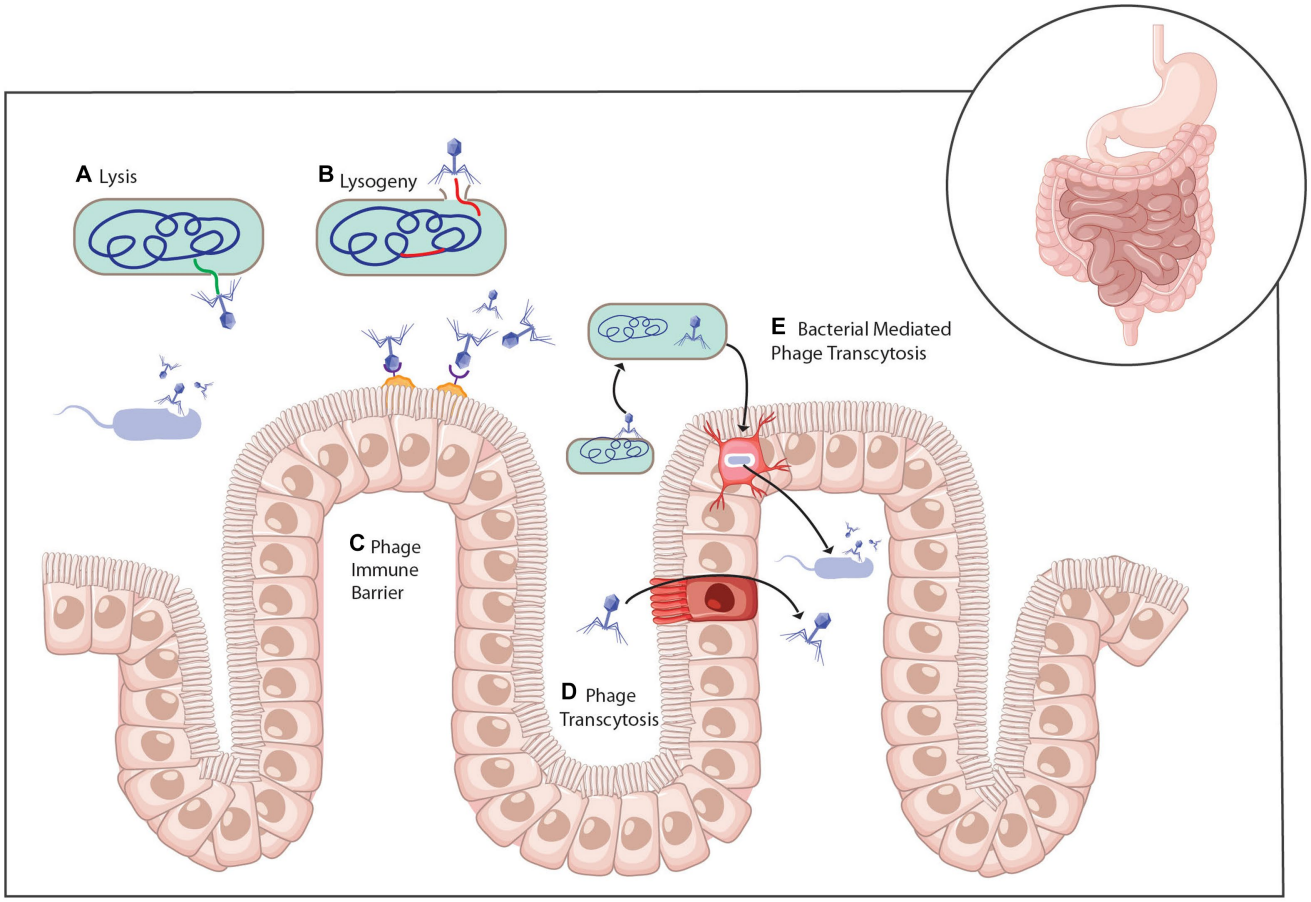
Mechanisms of phage interaction with bacteria and the mammalian host **(A)** Infection of bacteria with virulent phage leads to cell lysis and the release of progeny phage. **(B)** Temperate phage infection of a host bacterium can lead to the lytic life cycle or genomic integration into the bacterial chromosome as a prophage and the lysogenic life cycle. **(C)** Phage can attach to the mucosa through interactions with the capsid. **(D)** Phage can cross the intestinal epithelium through transcytosis or **(E)** within a bacterial cell (Baaziz et al., 2022).

The kind of interactions between phages and their bacterial hosts in the gut ecosystem have been described as highly complex (Zuppi et al., 2022). First, it is important to stress the fact that phages are host-specific and, as such, interact with one specific strain of bacteria. Because the gut harbors millions of microbial species, it is possible to have hundreds or even thousands of bacteria-phage interactions happening at the same time (Guinane and Cotter, 2013; Sausset et al., 2020). Second, the environment and conditions in the human gut create an enabling habitat that seems to protect or shield a fraction of bacterial species that are susceptible genetically to phage infections (Sausset et al., 2020). Third, it is possible to have both mutualistic and antagonistic interactions between phages and bacterial cells (Feichtmayer et al., 2017; Batinovic et al., 2019; Sausset et al., 2020). A typical example is predation, where bacterial cells get killed by their phage parasites following the lytic cycle (Figure 4). Contrarily, bacteria can interact with lysogenic or filamentous phages in a mutualistic way where both parties benefit one way or another (Van Belleghem et al., 2018; Naureen et al., 2020).

### 2.1. Predation

Phages often target bacterial prey through the recognition of some specific receptors on the bacterial surface membrane. Before the phage lysis, a bacterial cell first adsorbs (attaches) to susceptible hosts using its structural proteins (Xu et al., 2022). This phage action depends mainly on the complementarity between the molecular structures that serve as binding sites on the bacterial surface and the phage tail. The tail of phages possesses the capacity to hydrolyze the peptidoglycan cell walls of some bacteria using their lytic enzymes, which are responsible for releasing the phage progenies produced at the end of the lytic cycle. The peptidoglycan of the bacterial cell wall is disrupted and lysed by the lytic enzymes through the inhibition of processes and pathways resulting in the synthesis of peptidoglycans through a single protein or via peptidoglycan enzymolysis by utilizing lysin and other accessory systems (Xu et al., 2022).

There are two types of enzymes that can destroy the biofilms of bacteria. They are (i) exolytic enzymes responsible for promoting genome entry into bacteria cells in the early stages, and (ii) endolytic enzymes capable of degrading the bacterial host at the terminal stages to enable the phage progenies to be released into the immediate environment. Upon killing the gut bacteria via this unique process of predation, the gut phages may leave behind sequences of a specific CRISPR spacer on the genome of their victims. Through this sequence identification, bacterial death as a result of natural predation by phages, both in animals and humans, was discovered to be ubiquitous and function importantly in stabilizing the microbiota of the gut. The phage-bacteria predatory association is highly specific, as mentioned previously. An example was seen between phages of *Faecalibacterium prausnitzii*, which selectively infect their host, *F. prausnitzii*, ignoring other intestinal bacterial cells (Xu et al., 2022). However, an in-depth sequence of CRISPR spacer’s macrogenomic analysis disclosed that some phages in the gut of humans have a wider range of bacterial hosts (Xu et al., 2022). An example was seen when phages known to infect *F. prausnitzii* were found infecting and causing lysis in *Blautia hansenii*, which is only related from a distance to *F. prausnitzii* (Sausset et al., 2020).

Predatory interaction between phages and bacteria has been reported to drive co-evolution between the development of resistant mechanisms by the prey (bacteria) and a counter-effect by the predator (phages) (Koskella and Brockhurst, 2014; Braga et al., 2018; de Sordi et al., 2019; Nair et al., 2019). In some instances, a change in the genetic makeup of bacteria that can lead to resistance does not occur due to variation in their phenotypes (Baaziz et al., 2022). In some other instances, bacteria can completely remain susceptible to phage attack; however, the bacterial species often occupy regions deep inside the mucosa of the intestine, which is most often beyond the reach of phages under normal circumstances. The infectivity of bacterial cells by a phage virus has been reported to be reduced by some environmental conditions made possible by the GIT (Sausset et al., 2020). Reports have been made that the environment in the intestinal tract may be suitable to harbor some susceptible bacterial species in the absence of other resistance means, including phenotypic resistance (Corona and Martinez, 2013; Larsson and Flach, 2022). For instance, studies have demonstrated that phage infection of *E. coli* is impaired in a bile salt environment *in vitro*. The reason for this impairment has been attributed to phase-variable cell-surface protein repression (Kirsch et al., 2021). Furthermore, varying resistance by phages may occur due to a heterogeneous spatial population of bacteria induced via gradients of abiotic factors including oxygen and pH, as well as molecules like short-chain fatty acids, bile acids, and mucins (Sausset et al., 2020; Kirsch et al., 2021). Bacterial physiology can be modified by the aforementioned gradients, thus preventing successful phage predation. Furthermore, phage diffusion can be impaired through congestion of the GIT by varying non-compatible bacterial species, including other gut microbiota (Sausset et al., 2020).

### 2.2. Lysogenic conversion

Bacteria-phage interactions have extended not only beyond predation but also due to the potential benefits that lysogenic phages can confer on their bacterial hosts. Since a lysogenic phage can remain inactive/dormant in the bacterial host, its success in replication and survival is dependent on the host while it is still a lysogen. As such, an evolutionary advantage can be conferred on the bacterial host by the phage in an association that could be referred to as mutualism (Koskella and Brockhurst, 2014; Braga et al., 2018; de Sordi et al., 2019; Zuppi et al., 2022). This type of mutualism can be exemplified when virions carry genes that have no role or impact on the viral life cycle but are capable of conferring fitness benefits to their bacterial host. These genes are called morons, and the process through which they confer fitness to the bacterial host is known as lysogenic conversion (Zuppi et al., 2022). Temperate phages help the bacterial host adapt to adverse conditions present in the environment due to the expression of the newly acquired phenotypes (Davies et al., 2016; Lin et al., 2017; Rogovski et al., 2021; Xu et al., 2022; Marchi et al., 2023). These acquired phenotypes may contain traits responsible for bacterial immunity to other phage super-infections, resistance to antibiotics and other phages, pathogenicity, and tolerance to varying stresses, among other possible benefits (Sausset et al., 2020; Kirsch et al., 2021). Upon entering the bacterial host, lysogenic phages often integrate their genome with that of the bacterial chromosome. By doing so, the phage will be able to avoid being recognized and eliminated by the host’s macrophages, thus coexisting for a long time in the bacterial host (Xu et al., 2022). Aside from regulating the metabolism, abundance, and diversity of the gut bacterial communities, phages also serve as an important vehicle for horizontal gene transfer from one bacterial species to another.

### 2.3. Prophage induction

Prophages are stable inside the host; however, this stability can be distorted by stressors from the environment, and stochastic fluctuations can trigger the induction of phages, thereby causing them to resume the lytic cycle with the resultant lysis of bacterial host as described previously (Fortier and Sekulovic, 2013; Sausset et al., 2020). Generally, the induction of prophages is caused by cellular signals emanating from damages inflicted on DNA molecules through the destabilization of the master regulator or repressor proteins of lysogeny (Fortier and Sekulovic, 2013; Sausset et al., 2020). In the GIT, the prophage inducers that have been commonly associated with and described to cause the breakage of double strands of DNA are the quinolone antibiotics (Sausset et al., 2020). Fluoroquinolones and mitomycin C are common prophage inducers, in which case the bacterial RecA protein acts as a prophage response sensor (Henrot and Petit, 2022). On a similar note, carbadox, a prophylactic antibiotic, has been reported to cause the induction of prophages responsible for genes encoding Shiga exotoxin, which are transferred in cattle between strains of *E. coli* (Boling et al., 2020). As such, treatment of the infection of Shiga toxigenic *E. coli* in humans with quinolones has dire clinical consequences because they can destabilize the Stx prophages of *E. coli* (Sausset et al., 2020).

Evidence from various studies across the globe has suggested that rates of phage induction are said to be higher in the GIT of murine compared to classical growth cultures carried out *in vitro*, owing to an increase in the activation of the SOS response, which functions in the repair of damaged DNA. For *Lactobacillus reuteri*, however, the activation of the SOS response was suggested to be a result of activating specific metabolic pathways used in the GIT (Sausset et al., 2020). Sausset et al. (2020) also reported a possibility of a decrease or an increase in the induction rates of *E. coli* Stx prophage by metabolites in the gut such as bile salts or nitric oxide. Similarly, induction of *Salmonella* prophages by bile salts. However, inflammation of the intestine has been demonstrated to increase the induction rate of *Salmonella* prophages in a mouse model. Interestingly, the rate of phage induction has been reported to be regulated by the phages depending on the concentration of susceptible bacterial host communities in a newly discovered signaling pathway known as quorum sensing, especially in the phages of *Vibrio cholerae* and *Enterococcus faecalis* (Sausset et al., 2020). Combining these findings only points in a particular direction, suggesting that the induction of prophages might compound an immense burden on the bacterial host with great potential for altering the composition of the gut microbiota. Typically, this has been demonstrated using a mouse model; it was observed that a high rate of prophage induction modified the existing equilibrium between strains of bacteria by disfavoring the lysogens (Sausset et al., 2020).

It is worth mentioning that in the GIT, deoxyribonucleic acid (DNA) is actively being transduced by phages and transferred to other species or strains via horizontal gene transfer (Boling et al., 2020; Kirsch et al., 2021). Since the GIT allows for many of these events to take place, evidence has begun to emerge supporting the dynamic induction of prophages. For example, an axenic mouse was evaluated for the cost involved in carrying a prophage by *E. coli*. The result at the end of the experiment revealed that the rate of prophage induction was high (de Sordi et al., 2019). *In vivo*, prophage induction was also recorded for *E. faecalis*, where other competitors were observed to be killed (de Sordi et al., 2019). Studies of *E. faecalis* revealed also that they have intricate behaviors of varying elements of prophage all within the same cell, as some of the defective prophages were observed to hijack some structural proteins present in intact prophages to be able to form virion necessary for their dissemination into the surrounding environment (de Sordi et al., 2019).

## 3. Ecological models and dynamics of phage-bacteria interaction

Understanding the interactions between phages and their bacterial host is paramount if they should be considered an important therapeutic or diagnostic tool in the near future. As a result, several ecological models have been postulated and utilized to demystify the interactions between phages and their bacterial host in a biological system context (i.e., the gut of humans) (Sutton and Hill, 2019). However, Zuppi et al. (2022) were able to categorize these models into two broad groups based on their unique features.

### 3.1. Group 1 models

Models in this group are characterized by low diversity and variability of both bacteria and phage populations [i.e., Piggyback-the-Winner and Arms-Race Dynamic (ARD)] (Zuppi et al., 2022).

#### 3.1.1. The arms-race dynamic

The need for colonization and survival is necessary for any microorganism to be successful in any given ecosystem. As such, they must compete for the necessities present in the habitat. This interaction causes both genotypic and phenotypic changes in the competitors for the microorganism to adapt well to stressors (Fortuna et al., 2019). Bacteria (prey) have defense mechanisms against phage predation, and phages (predators) have the means to evade prey defenses (Fortuna et al., 2019). As described, this dynamic is coevolutionary, which was previously mentioned. This defense and counterdefense may lead to an arms race between phages and bacteria where respective species that are not capable of withstanding this barrier will go extinct, leaving the ones that are competent to flourish (Scanlan, 2017; Zuppi et al., 2022). The ARD model sees both competitors (bacteria and phages) accumulate varying numbers of mutant genes in their genomes, which enhances the evolution of resistance by the bacteria to counteract the infection by the phage and, in the process, continues to generate the predator–prey cycles (de Sordi et al., 2019). In the ARD, there is a selection toward the development of an ever-increasing counterdefense by parasites against the host defense (Fortuna et al., 2019). Mutations in the ARD model can restore phage infectivity against bacteria, resulting in a predator–prey cycle in which bacteria and phages evolve constantly to avoid being eradicated from the gut microbiome (Sutton and Hill, 2019; Dahlman et al., 2021; Zuppi et al., 2022). So far, it is still early to conclude on the extended ARD within the GIT owing to limited evidence; nonetheless, phage resistance has been reported (Gilpin and Feldman, 2017; Sausset et al., 2020; Spencer et al., 2022). Moreover, a great diversity of microorganisms has been reported to assist in transitioning from one host to another, and lytic coliphages have been hypothesized to survive in the guts of infants. However, it is yet to be seen in the stable gut of an adult the applicability of this process. Rather, evidence from recent studies suggests that the existence of spatial heterogeneity within the gut (i.e., the mucosal surface vs. the lumen surfaces) is a potential driver of this coexistence between the phage and the host (Dahlman et al., 2021).

#### 3.1.2. The piggyback-the-winner dynamic

Mutualistic interaction is the main driver for this model, and it mainly occurs in the life cycle of lysogenic phages. In a real sense, this model describes the contribution made by prophages to the survival of bacterial hosts by protecting them from reinfection by other phages, a process known as superinfection exclusion (Kirsch et al., 2021; Zuppi et al., 2022). Due to the added advantage provided by the lysogens, the bacterial host is being conferred traits that enable it to persist in a competitive environment. By doing so, both the phages and their host prolong the gut ecosystem, thanks to the mutual relationship created. Most importantly, it is worth mentioning that the proposed piggyback-the-winner dynamic (PtW) model is the only model of population dynamics between bacteria and phages that is directly linked to the lysogenic cycle (Voigt et al., 2021; Zuppi et al., 2022). Models such as PtW that allow for the switch from a lytic cycle to a lysogenic mode of replication also support the dynamics of phage-host interaction, with emerging experimental evidence of applicability within the GIT of mammals (Sutton and Hill, 2019). A reduced ratio of bacteria-to-phage population could best be described when temperate phages enter lysogeny. Most gut bacteria have been reported to be lysogenic by a minimum of one temperate phage (Kirsch et al., 2021). The mucosal surface of the GIT has been demonstrated to support PtW model interactions between phages and their hosts. The surface of the mucosa has been suggested to have high colonization by bacterial populations. Thus, they contain a high virus-to-microbe ratio (VMR), which gives rise to PtW dynamics. However, the deeper layers of the mucosal membrane have been reported to support the model known as Kill-the-Winner (KtW) (Sutton and Hill, 2019).

### 3.2. Group 2 model

This group contains models that are characterized by high diversity and variability of bacteria and phages (kill-the-winner dynamics and fluctuating selection dynamics) (Zuppi et al., 2022). The driving force behind these models is selection dependent on negative frequency; it is a natural form of selection where a genotype’s fitness is inversely proportional to its frequency (Koskella and Brockhurst, 2014; Scanlan, 2017; Braga et al., 2018; Brisson, 2018; Zuppi et al., 2022).

#### 3.2.1. The fluctuating-selection dynamic

This model is also known as Red Queen Dynamics (Fortuna et al., 2019). The model explains the interactions between bacteria and phages; the relationship has been proposed to be a parasitic one where phages attack bacteria and, in return, experience counter-resistance from the prey (Kirsch et al., 2021). Unlike the Arms Race model, which results in rapid gene evolution but generally low levels of standing genetic diversity, Red Queen dynamics result in balanced polymorphisms with long coalescence durations (Koskella and Brockhurst, 2014). The model of fluctuating-selection dynamic (FSD) considers the associated pleiotropy with respect to mutations, which enables bacteria to be resistant to attack by phages; as such, the phages do not depend on phage evolution (de Sordi et al., 2019). In the FSD model, phages infect hosts that have resistance traits and, at the same time, reduce the number of phage virions present in the environment (Sutton and Hill, 2019). The reduction in the number of resistant prey allows for the proliferation and expansion of susceptible strains of bacterial prey. As such, the abundant susceptible prey now has a competitive advantage over the resistant host, which, as a result, deprives them of space and nutrients, eventually making them go extinct (de Sordi et al., 2019; Sutton and Hill, 2019). This transient infectivity and resistance of phage-bacteria communities often result in short-term fluctuations in the number of bacteria, with both persisting for a long time (Sutton and Hill, 2019). The FSD model was a result of the fitness cost needed by the bacteria to develop mechanisms to resist phage attacks, which is quite difficult to achieve, especially in an environment (such as the gut) harboring varying species competing at the same time for resources (Zuppi et al., 2022). The number of infective phages is set to decrease due to an effective mechanism that helps in resisting phage infectivity. The population of bacteria resistant to phages may be outcompeted if they have low numbers when the rate of predation decreases. As a result, bacteria that are not resistant to phage infectivity will make up the majority of the bacterial communities. As phage predation rises in the newly generated permissive environment, the environment becomes once more conducive to the evolution of bacteria that are resistant to phages, thereby increasing the overall number of resistance phages. Consequently, the cycle starts afresh and the process repeats itself (Colavecchio et al., 2017; Lin et al., 2017; Oechslin, 2018; Zuppi et al., 2022). Under the FSD model, genotypes of the host that are uncommon are favored by natural selection only if they can escape phage attacks that are adapted locally to the genotype that is the most common. Concurrently, the selection process will continue, whereby phages able to attack the commonest of hosts are favored due to the specificity of the phage-host relationship (Fortuna et al., 2019). The FSD model applies to pairs of bacterium-phages as well as the mixed population developed through convolution (de Sordi et al., 2019).

#### 3.2.2. The kill-the-winner dynamic

The interactions between bacteria and phages have also been explained using kill-the-winner (KtW) dynamics (Kirsch et al., 2021). This model is said to be analogous to the Lotka-Volterra equation (Bacaër, 2011), which describes the interactions between predators and their prey (Kirsch et al., 2021). The density of the bacterial population is the key determining factor in the KtW model of a non-temperate lytic phage (Kirsch et al., 2021). Dominant and fast-growing commensals are the first to be targeted and killed by phages before the slower-growing prey, reducing their population in the human gut because the movement of active phages toward a host is quite slow. As such, the KtW model is dependent on the bacterial population of interest being temporarily high within the population of phages as they wait for any opportunistic contact with the bacterial host for infectivity and replication (Mukhopadhya et al., 2019).

A host-phage interaction is said to be low when the bacterial growth is low, thereby resulting in a low rate of infectivity. When the population of bacterial hosts expands, however, the probability of a phage coming into contact with a host also increases (Kirsch et al., 2021). The expansion of the bacterial population is hampered when there is a higher rate of phage-bacteria infectivity. Due to this, phages can act as a stabilizer in the KtW model to stabilize the composition of bacterial communities in a biased manner that prevents the fast-growing species of bacteria from overrunning the population of the community (Zuppi et al., 2022). Multiple competing phages may not simultaneously infect multiple bacterial lineages due to phage-host specificity (Myers et al., 2023). A sudden collapse of the microbial community can be observed when the KtW model is applied to the bacterial host infected by virulent phages. In a highly heterogeneous bacterial community, each species observed a cyclical population dynamic, whereby an expanded population collapsed abruptly. However, population diversity is always maintained, provided the bacterial community grows at a high rate relative to the composition of bacterial communities. For instance, the KtW model is applicable in the gut microbial community of humans, with the notable exception of bacteria-phage interactions in the gut of infants (Kirsch et al., 2021). As such, the KtW model is considered only for microbial species that are dominant in a community with a paucity of data regarding the occurrence of this model in the human gut. Even contrived models stacked with a single strain of *E. coli* alongside infection by coliphage failed to reveal the applicability of this phenomenon. The possibility of the physiological nature and physical structure of the gut environment protecting the bacteria against coming in contact with phages has been suggested to be the stumbling block limiting the applicability of this model in the gut of humans (Mukhopadhya et al., 2019).

## 4. Bacteriophage activities in the GIT in relation to health and disease

Advances in high-throughput technologies used in sequencing have made the GIT virome to be considered a vital component of the microbiome of the human gut, where the overall virome is believed to outnumber and outcompete the population of bacteria (Zuo, 2018). With the revelation about the gut virome via high-throughput sequencing, there is a paucity of data about their ecological roles and functions in influencing the health of humans. It is no longer news that pathogenic viruses in the guts of humans inflict deleterious effects (Zhang et al., 2015; Lecuit and Eloit, 2017). Interestingly, however, there are numerous instances where they confer traits and effects that are beneficial to gut bacterial species via different mechanisms and interactions; they can also modulate the adaptive and innate immune systems of the host (Zuo, 2018). Due to this, viruses (particularly phages) have been utilized for diagnostic and therapeutic purposes (Brives and Pourraz, 2020).

### 4.1. Therapeutic potential of bacteriophages

The global healthcare system is currently being challenged by antibiotic-resistant strains of microorganisms, thus causing researchers across the globe to search for new modified antibiotics (Loganathan et al., 2021; Shuwen and Kefeng, 2022). However, it is a daunting task to search for new antibiotics, which may take numerous years to achieve. Pathogenic bacteria that cause GIT infections (such as *Salmonella*, *Shigella*, *Campylobacter jejuni*, *E. coli*, *Clostridium difficile*, and *Vibrio cholera*) have the ability to cause chronic or even acute infectious enteritis and have consistently demonstrated resistance to newly developed antibiotics (Xu et al., 2022). Since phages have the ability to lyse pathogenic bacterial cells via their lytic cycle, they have been proposed to be an important therapeutic agent in the fight against the global pandemic of antibiotic resistance in a process known as phage therapy (Sulakvelidze et al., 2001; Shuwen and Kefeng, 2022). Phage therapy is simply the use of bacteriophages in the treatment of diseases caused by bacteria (Brives and Pourraz, 2020; Baron, 2022); it is a novel therapeutic increasingly gaining interest by clinicians in treating infections that are resistant to multiple drugs (Baron, 2022). Phage therapy has also been considered an important adjuvant in treating chronic biofilm infections (Doub, 2021). However, some conditions/criteria need to be met before initiating phage therapy for any disease condition(s).

### 4.2. Criteria considered for phage therapy

It is pertinent that phages employed as therapeutics are devoid of genes encoding toxin proteins or virulence factors (Manohar et al., 2019). A lysogenic phage (i.e., phages that integrate into the host chromosome) should be critically considered before use, since they can increase the virulence of the pathogen by conferring more traits (such as toxin production or even resistance to more classes of antibiotics) that will require another specific virulent phage to lyse the pathogenic bacteria. As such, it is more recommendable to use lytic phages since they directly cause the lysis of targeted pathogenic bacteria cells (Manohar et al., 2019). How effective phage therapy will become is strongly dependent on the ability of the phage to overcome the barrier posed by the epithelium and induce the immune system to respond adequately (Sinha and Maurice, 2019). However, the potential for pro-inflammatory (promoting inflammation) responses and antibody production by the human immune system has raised questions about the safety and efficacy of phage therapy. As such, it is pertinent to have an in-depth understanding and knowledge of the particular phage taxa that is considered for therapy to prevent any form of anti- or pro-inflammatory response (Sinha and Maurice, 2019). Therefore, getting a suitable phage is a prerequisite for initiating any form of phage therapy in any disease condition. This can be achieved by performing preclinical studies both *in vivo* and *in vitro* to ascertain if the phage fulfills the criteria to be used for therapeutic purposes (Manohar et al., 2019).

### 4.3. Phages and the immune response of humans

Immunological responses by the human body owing to phage exposure via oral route or injection into the blood are vital aspects of phage therapy (Manohar et al., 2019). The immune system of humans is a complex network that reacts whenever any foreign particles (including viral particles) enter the body. As mentioned previously, phages are important components of a healthy human virobiota/microbiota with numerous studies being conducted to ascertain the roles they play in the gut microbiome (Manohar et al., 2019). However, the presence of phages may not be harmful to the human body because they are not often identified as being noxious by the immune system (Loc-Carrillo and Abedon, 2011). Explicit evidence is yet to be demonstrated concerning an elicit response by the immune system toward phages, including the elimination of other microbial viruses. By size comparison, eukaryotic viruses are much smaller than phages, yet there is a dearth of knowledge on how phages interact and induce the immune system (Manohar et al., 2019). For example, animal or human immunization with phages has been reported to produce antisera against phages (i.e., antibodies that act against phage proliferation), with the non-immunized animals or humans having the antisera only at a very low concentration (Manohar et al., 2019). The suggestion has also been made regarding the presence of natural antibodies for T-like phages as a result of their common presence in the normal flora of the human gut. In the circulatory system, however, the presence of phages is not welcomed by the human immune system. For example, the mutant lambda phages that were inoculated into the circulatory system of an experimental model saw the innate immune system eliminate the phages with their circulating time in the blood reduced.

The immune system of the body was triggered to act against the inoculated phages. Studies that can demystify the mechanism behind cellular responses against phages are few. Moreover, there is current controversy over the manner in which phages are being presented by antigen-presenting cells to the T-cells (for initiating the production of antibodies and future memory responses). Studies have been done, but there remains substantial research to establish the precise mechanism of the interaction of phages with the immune system of humans. Although humans have been conducting numerous studies on the phage-immune system interaction, human knowledge and understanding regarding this subject remain to be fully understood (Manohar et al., 2019).

The potential of phages to affect the immune response is another interesting characteristic of phages. It has been highlighted in this review paper how phages actively scavenge and parasitize invasive bacteria, reduce inflammatory and other immune responses, and maintain a stable gut microbiota (Xu et al., 2022). Lysis mediated by phages has been reported to be involved not only in pathogen-associated molecular patterns (PAMPs) but also in the translocation and activation of immune responses whenever there is an increase in the permeability of the intestine. Pathogen-associated molecular patterns (PAMPs) are molecules with highly conserved structural motifs produced by microorganisms and detected by pathogen recognition receptors (PRRs) as foreign to initiating immune responses (Silva-Gomes et al., 2014; Asiamah et al., 2019). Through the inducement of macrophages by phages, bacteria are being phagocytized through opsonization (an immune process and a means or molecular mechanism to dispose of antibody-coated antigens that are small enough to be engulfed by a phagocyte) to facilitate easy entrance into the immune system, where they are destroyed. It is important to note that an important determining factor between phages and their bacterial hosts is the intestinal mucosa (Łusiak-Szelachowska et al., 2017; Scanlan, 2020; Xu et al., 2022).

The phage communities sometimes establish contact with the mucosal barriers and generate an immune response. This way, commensal microorganisms get protected by innate immunity so long as they are located at the upper layer of the mucus membrane, while the acquired immunity kills pathogens that invade the deepest layer of the mucus through lysis (Xu et al., 2022). To effectively play the role of an antibacterial agent, phages adherent to the mucus membrane prevent bacterial colonization. Some phages do express proteins that display domains type immunoglobulin and the immune folds of C-type lectin, which interfere with O-glycosylated MUC2 expression by mucin in the colon. On the contrary, pathogens that can disrupt responses through innate immunity will in turn be taken care of by the acquired immune accessories (Xu et al., 2022). Factors in the intestine responsible for limiting phages have been considered to be immunoglobulin A (IgA) production.

The key factor limiting phage activity in the stomach appears to be phage-specific secretory IgA. Notably, after the phage was eliminated from the diet, secretory IgA levels declined over time. There was no substantial rise in anti-phage IgM antibodies, and no phage-specific IgA antibodies were discovered in the blood (Majewska et al., 2019). According to Xu et al. (2022), phages are discovered in feces when IgA levels are low, but when IgA levels are high, no active phages are identified in feces, which may also directly explain the connection between the relative abundance of intestinal phages and IgA-related immunity. This strongly suggests that IgA has a profound effect on phages present in the gut of humans (Xu et al., 2022).

Phage interactions with mammalian/immune cells are extensively studied, encompassing phage immunomodulation and immunogenicity. In the development of vaccines by various researchers across the globe, phages have been employed as vehicles to achieve adequate delivery. Reports have been made regarding the usage of FD phages to stimulate responses by dendritic cells, which activate both adaptive and innate immune systems as the viral particles are perceived as pathogens (Manohar et al., 2019). The fd phage is a filamentous virus whose structure is formed by a cylindrical flexible protein scaffold, about 7 × 890 nm, containing a single-strand DNA genome rich in CpG motifs (Prisco and De Berardinis, 2012). Individuals suffering from immunodeficiency mediated by impaired antibody responses are said to be alleviated using phages during immunization (Manohar et al., 2019). Phages can also have a negative impact on the immune system. For example, *E. coli* Stx prophages have been found to carry genes encoding for Shiga toxin, which can modify the innate immunological response of human enterocytes by inhibiting the PI3K/Akt/NFB signaling pathway. This, in turn, reduces the levels of interleukin-8 and the chemokine CCL20 (Davies et al., 2016).

### 4.4. Strategies for phage therapy

In theory, all bacteria can be lysed by at least one or more phages. As such, phages have been considered an important weapon in the fight against bacterial strains resistant to multiple antibiotics. During therapy, the number of phage types is used to characterize phage therapy as either monophage or polyphage, where the former employs only one type of phage while the latter utilizes more than one type of phage. In practice, the use of a combination of more than one phage in therapy is referred to as a “phage cocktail” (Chan et al., 2013). The use of monophage therapy is often employed during experimental trials, especially to prove a concept or develop models, such as scenarios where phages capable of infecting a wide range of hosts are available or in a clinical setting where exact matching of certain phage isolates and pathogens is necessary (Chan et al., 2013). Although broad-spectrum antibiotics exist, they cannot kill all strains of bacteria. Moreover, the use of broad-spectrum antibiotics is not recommendable due to the deleterious impact they can have on the normal microbiota of the human body. This is not the case with phages since they are host-specific and, as such, eliminate one of the problems caused by broad-spectrum antibiotics (Principi et al., 2019).

### 4.5. Phage therapy in the treatment of gastrointestinal diseases

Bacterial infections common to the GIT include diarrhea, dysentery, gastroenteritis, cholera, and salmonellosis caused by *C. difficile*, *Shigella*, *E. coli*, *V. cholerae*, and *Salmonella enteritidis*, respectively. Phage therapy has been reported to restore the balance of the gut microbiota. A synergistic effect has been proposed to be the reason why a cocktail of phages is regarded as more effective than monophage therapy. Shuwen and Kefeng (2022) reported a clinical treatment involving a 68-year-old patient who was suffering from necrotizing pancreatitis alongside infection with *Acinetobacter baumannii*, which was resistant to multiple antibiotics during treatment. Since treatment with antibiotics was futile, researchers decided to isolate *A. baumannii*, which was screened for susceptibility to phage cocktails. Eventually, a cocktail of phages capable of causing lysis in the antibiotic-resistant bacteria was injected intravenously into the patient. Rapid improvement was then observed in the patient following treatment (Shuwen and Kefeng, 2022). An enteropathogenic *Escherichia coli* (EPEC)-infected mouse model was used to investigate the effect of ciprofloxacin and phages to determine which of the therapies is more efficient and successful as a therapeutic agent. It was also observed that phage therapy was not only effective *in vivo* in reducing the number of EPEC but also sufficient to cause normal growth in the mice. Similarly, an investigation on a rabbit model infected with *V. cholerae* was done to ascertain the efficacy of the phage cocktail (Xu et al., 2022). After 6 h of treatment with the phage cocktail, it was observed that the proliferation of *V. cholerae* was significantly reduced in the rabbit model used. This outcome was therefore correlated to the time of phage cocktail administration (Xu et al., 2022).

### 4.6. Advantages of phage therapy

The use of phage for therapeutic purposes has several advantages over the use of conventional antibiotics owing to their properties and the options they provide to clinicians (Loc-Carrillo and Abedon, 2011). Depending on the densities of bacterial species, phages, during the lysis of the bacterial host, have the ability to multiply in large numbers, especially in places where the bacterial host is located, a phenomenon known as “auto dosing,” since the phages are the ones responsible for this large increase in phage populations within a short period of time (Loganathan et al., 2021). Although reports have been made that phages are specific in the type of host they infect. Nevertheless, reports have been made where phages were able to infect other closely related bacterial species (Loc-Carrillo and Abedon, 2011). This is in contrast to conventional antibiotics, where they have been reported to command a broader range of antibacterial activity, thereby promoting superinfections such as yeast infections caused by *Candida albicans* or colitis caused by *C. difficile*. The fact that phages have a relatively narrow range of hosts has made selection for the development of a phage-resistant mechanism limited to specific phage species (Loc-Carrillo and Abedon, 2011; Loganathan et al., 2021). For example, collateral damage was not recorded in other bacterial communities when a mouse model infected with diarrhea-causing *E. coli* was treated with T4-like phages (Principi et al., 2019).

Bacteria that form biofilms tend to have added advantages in terms of resisting the actions of antibiotics. Phages, on the other hand, have been demonstrated to possess the ability to penetrate (i.e., penetrating one bacterial cell layer at a time through lysis) or clear bacterial biofilm using depolymerases and exopolymer-degrading enzymes (Loganathan et al., 2021). Like antibiotics, phages are versatile as they can be formulated into varying forms (i.e., impregnated in solid materials, added to creams, or even formulated into liquid) during development. They can also be used together with other therapeutics (i.e., antibiotics). Phages can also be formulated in such a way that they are applicable via various routes (i.e., oral, inhalation, or intravenous) (Loc-Carrillo and Abedon, 2011). Furthermore, lytic phages have been identified to be very effective within a given short period of time (Loganathan et al., 2021).

### 4.7. Limitations of phage therapy

Although phages are ubiquitous, not all can be used for therapeutic purposes. The most common types of phages used for therapy are lytic phages. The applicability of phages in therapy has raised a lot of questions, especially the problems associated with purity and safety, as some phages have been reported to encode for toxins. In the preparation of phages, if care is not taken, there is the possibility that the phage prepared may possess endotoxic proteins acquired from the bacterial host (Shuwen and Kefeng, 2022). Furthermore, the lysis of the bacterial host can release endotoxin into the human gut, which can be deleterious; specifically, while solving a particular problem at the end, a different problem is created (Shuwen and Kefeng, 2022). While it has been stated earlier that polyphage therapy is highly effective, nevertheless, with the use of FMT during therapy, other important normal flora can be eliminated from the microbiota of the gut. This also raises concern, as the whole desire is to eliminate only the desired harmful bacterial species (Cao et al., 2022). Once administered to patients, the safety of the host inside the patients is not completely certain, as phages have been demonstrated to be an important gene-editing tool that can evolve easily and cause deleterious effects when they interact with the human immune system (Shuwen and Kefeng, 2022).

In addition, phages that use the lysogenic cycle have been identified to have the ability to integrate their genome into the chromosome of the bacterial host. In scenarios where they possess antibiotic-resistant genes, they will only go on to cause further harm to the patient undergoing treatment since an urgent need will arise to eliminate the antibiotic-resistant bacterial species (Loganathan et al., 2021). Since phages have a host, they are effective against as such, in scenarios where a prepared phage cocktail does not contain phages that can infect and cause lysis to target bacteria, the therapy will end up not being effective (Gutiérrez and Domingo-Calap, 2020).

## 5. Conclusion and future research

Phages are an important group of gut microbiota whose roles are still not clear in inducing or regulating the immune response of humans. Nevertheless, phages have shown promising potential to be used as an effective therapeutic tool, especially in the treatment of intestinal disorders. Future research is expected to increase understanding of how bacteriophages influence the variety and composition of bacteria in the human gut and how they might be used to treat diseases. Scientists anticipate better knowledge of how bacteriophages modify the gut microbiota, affecting human health and illness, as research develops. Interactions between bacteriophage and bacteria in the gut ecosystem are anticipated to be more thoroughly characterized owing to advances in developing technology and computational methods. This will result in the creation of customized phage treatments, a potential replacement for conventional antibiotics, which may selectively eradicate multi-drug-resistant bacteria while protecting good ones. Furthermore, taking into account variables like genetics, nutrition, and lifestyle, individualized phage-based therapies may be developed to address individual variances in gut microbiomes.

Phages may become more precise and successful gut microbiota therapies when their full potential is realized, resulting in better health outcomes for people all over the world. Before such treatments are widely used, thorough studies and clinical studies will be required to confirm their safety and effectiveness. As such, more studies involving experimental models and clinical trials are needed to widen the understanding of bacteria-phage interactions and their association with immunological responses in the human gut.

## Author contributions

SSA wrote the manuscript, prepared the figures, and approved the submitted version.

## Conflict of interest

The author declares that the research was conducted in the absence of any commercial or financial relationships that could be construed as a potential conflict of interest.

## Publisher’s note

All claims expressed in this article are solely those of the authors and do not necessarily represent those of their affiliated organizations, or those of the publisher, the editors and the reviewers. Any product that may be evaluated in this article, or claim that may be made by its manufacturer, is not guaranteed or endorsed by the publisher.
